# Supplementary material for: A Label-Free Cell Sorting Approach to Highlight the Impact of Intratumoral Cellular Heterogeneity and Cancer Stem Cells on Response to Therapies
Source: Cells. 2022 Jul 22;11(15):2264. doi: 10.3390/cells11152264 (PMC9332486; doi:10.3390/cells11152264)
Supplement: Supplementary file 1 [file cells-11-02264-s001.zip › cells-1794666-supplementary.pdf]

Supplementary files

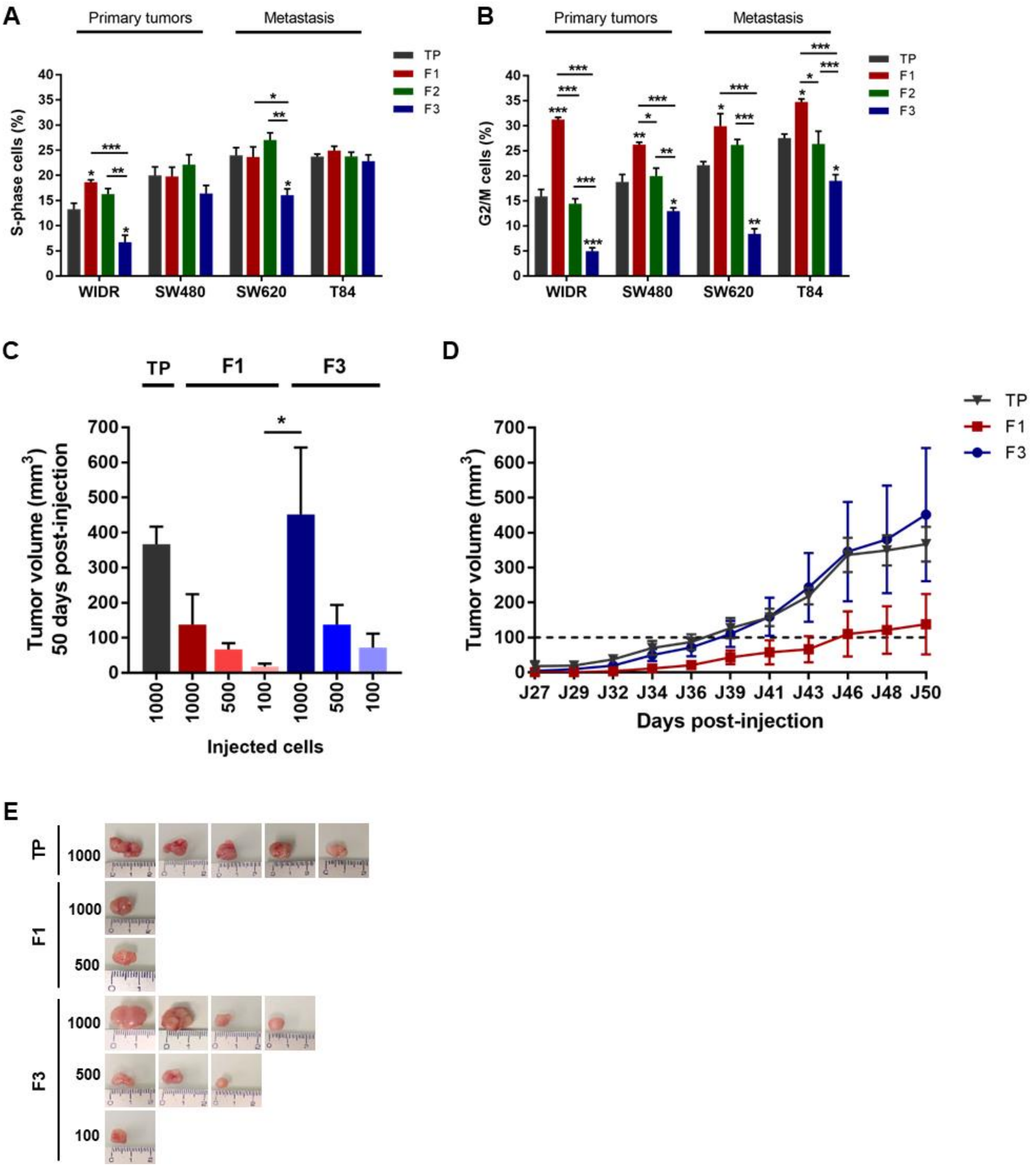

Figure S1. Phenotypic and functional characterization of sedimentation field-flow fractionation (SdFFF)-sorted cell subpopulations from colorectal cancer (CRC) cell lines. (A-B) The proportion of S-phase and G2/M cells in the cell cycle was analyzed by flow cytometry for each sorted cell subpopulation and presented in the bar plot from at least three biological replicates. (C-D) Tumor volume was measured throughout the in vivo tumor initiation assay using a caliper. (C) Fifty days after injection, the average tumor volume obtained in the five mice in each cell concentration group is summarized in the bar plot. (D) The appearance of a tumor with a volume greater than 100 mm3 was evaluated using tumor growth curves. (E) Collected tumors larger than 100 mm^3 were photographed 50 days after injection. Scale in centimeters. All these results are represented as means ± SEM and statistical differences with *p-value < 0.05, **p-value < 0.01, ***p-value < 0.001 and *alone for significant results compared to TP using One-way ANOVA test.

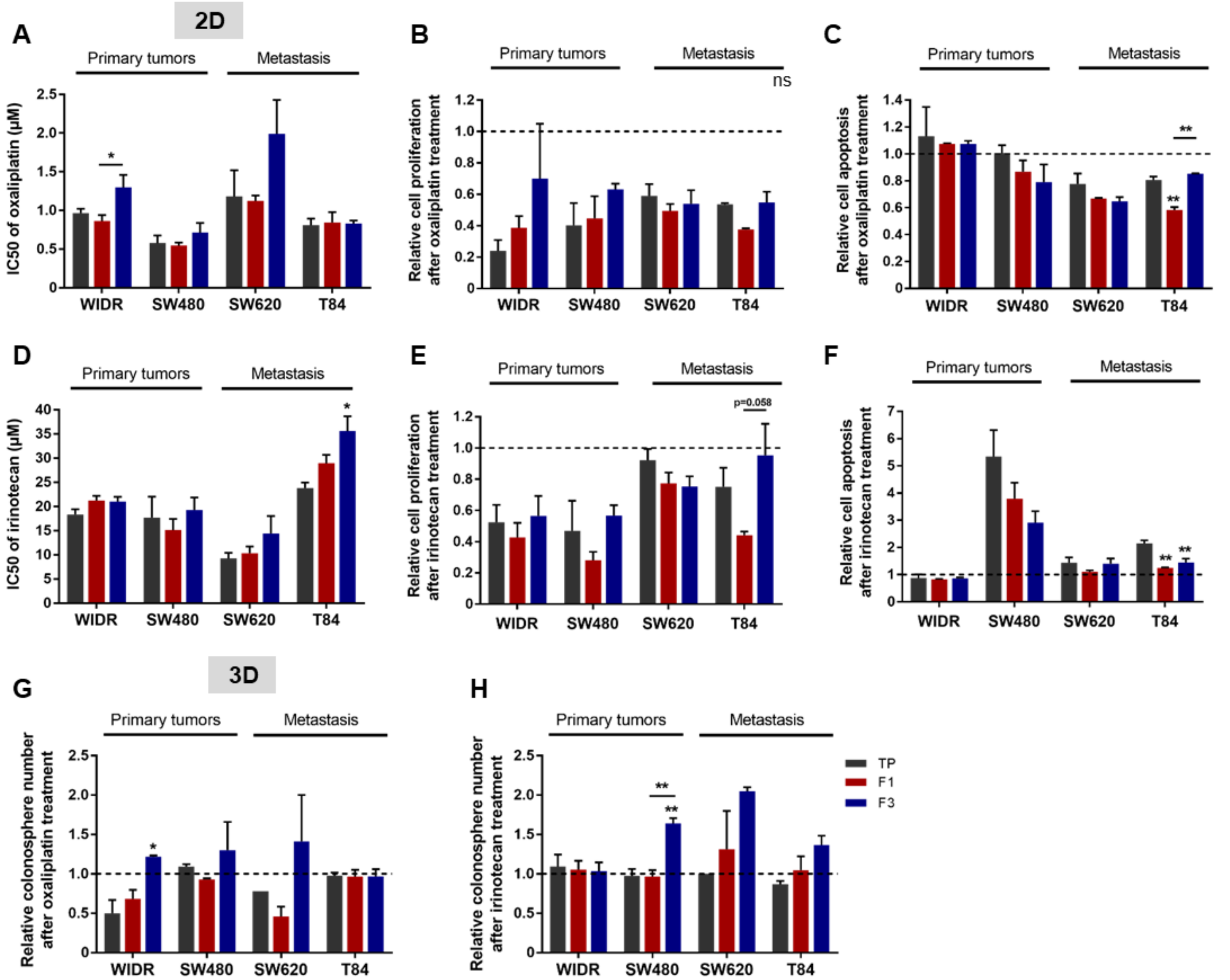

Figure S2. Response of SdFFF-sorted cell subpopulations to oxaliplatin and irinotecan from CRC cell lines. (A-F) Response to oxaliplatin and irinotecan was assessed in 2D culture. (A) After three days of oxaliplatin treatment, IC50 values were obtained by MTT assay from at least three biological replicates. (B) Cell proliferation rate after oxaliplatin treatment was measured by BrdU assay and presented in the bar plot as a ratio between treated and untreated conditions (dashed line). (C) Using the ELISA cell death assay, apoptosis rate after treatment was measured and compared to the untreated condition (dashed line). (D) After three days of irinotecan treatment, IC50 values were obtained by MTT assay. (E) As with oxaliplatin, cell proliferation was assessed after irinotecan treatment and compared with the untreated condition. (F) Apoptosis was also assessed after irinotecan treatment. (G-H) Response to oxaliplatin and irinotecan was also investigated in 3D culture from colonospheres. The number of colonospheres was assessed after treatment with oxaliplatin (G) or irinotecan (H). All these results are represented as means ± SEM and statistical differences with ns means a non-significant result both between the sorted cell subpopulations and between a sorted subpopulation and the control, *p-value < 0.05, **p-value < 0.01 and *alone for significant results compared to TP using One-way ANOVA test.

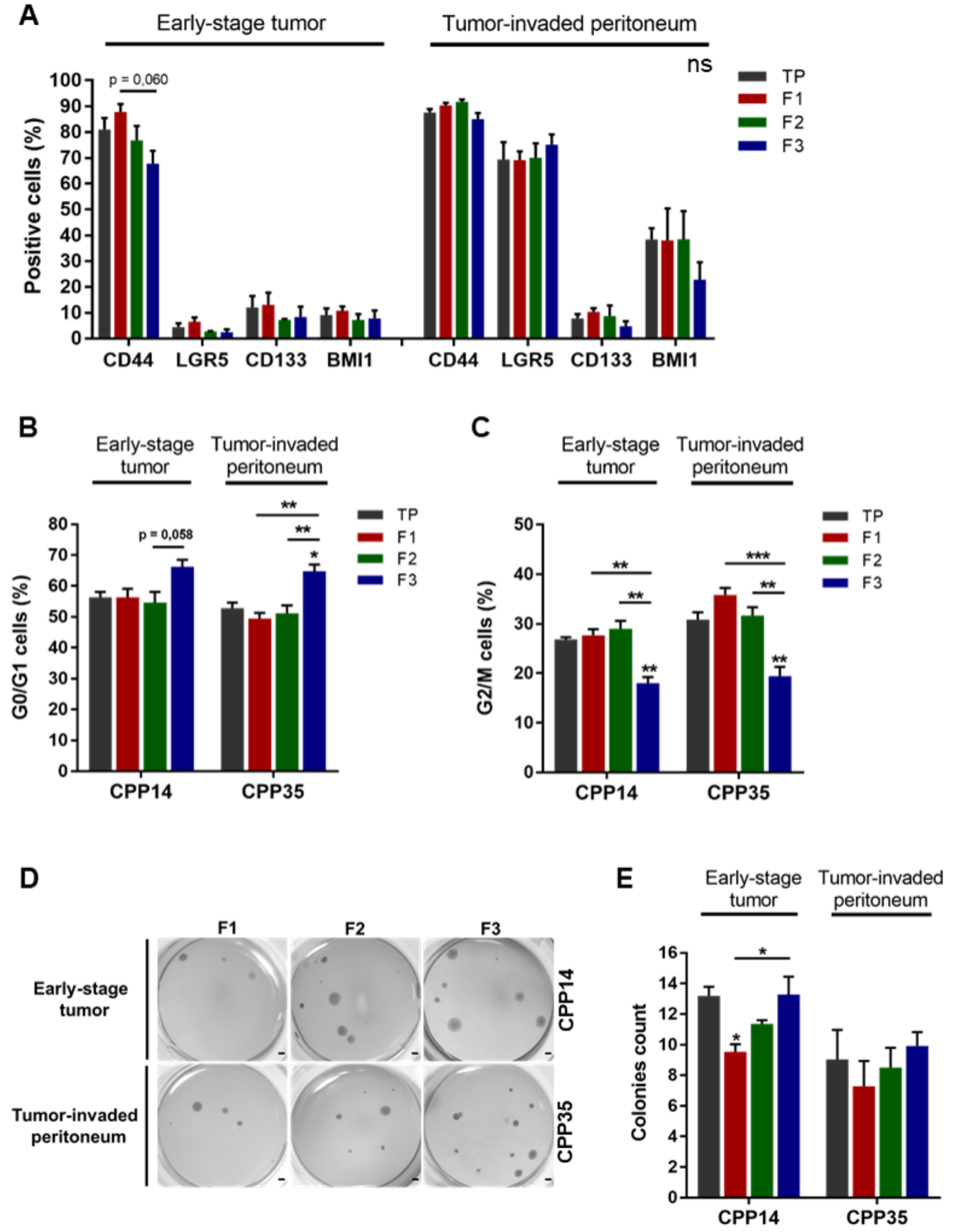

Figure S3. Phenotypic and functional characterization of SdFFF-sorted cell subpopulations from CRC primary cultures. (A) The expression level of CSC markers, CD44, LGR5, BMI1, and CD133, was assessed by flow cytometry and plotted as a bar plot from three biological replicates. (B-C) Cells in G0/G1 phase (B) or G2/M phase (C) were quantified by flow cytometry. (D-E) Cell clonogenicity was assessed by a soft agar assay and presented as images of the colonies formed (D) as well as their quantification in the bar plot (E). Scale bar 1 mm. All these results are represented as means ± SEM and statistical differences with ns means a non-significant result both between the sorted cell subpopulations and between a sorted subpopulation and the control, *p-value < 0.05, **p-value < 0.01, ***p-value < 0.001 and *alone for significant results compared to TP using One-way ANOVA test for analysis of CSC marker expression and cell cycle distribution, and Kruskal-Wallis test for clonogenicity.

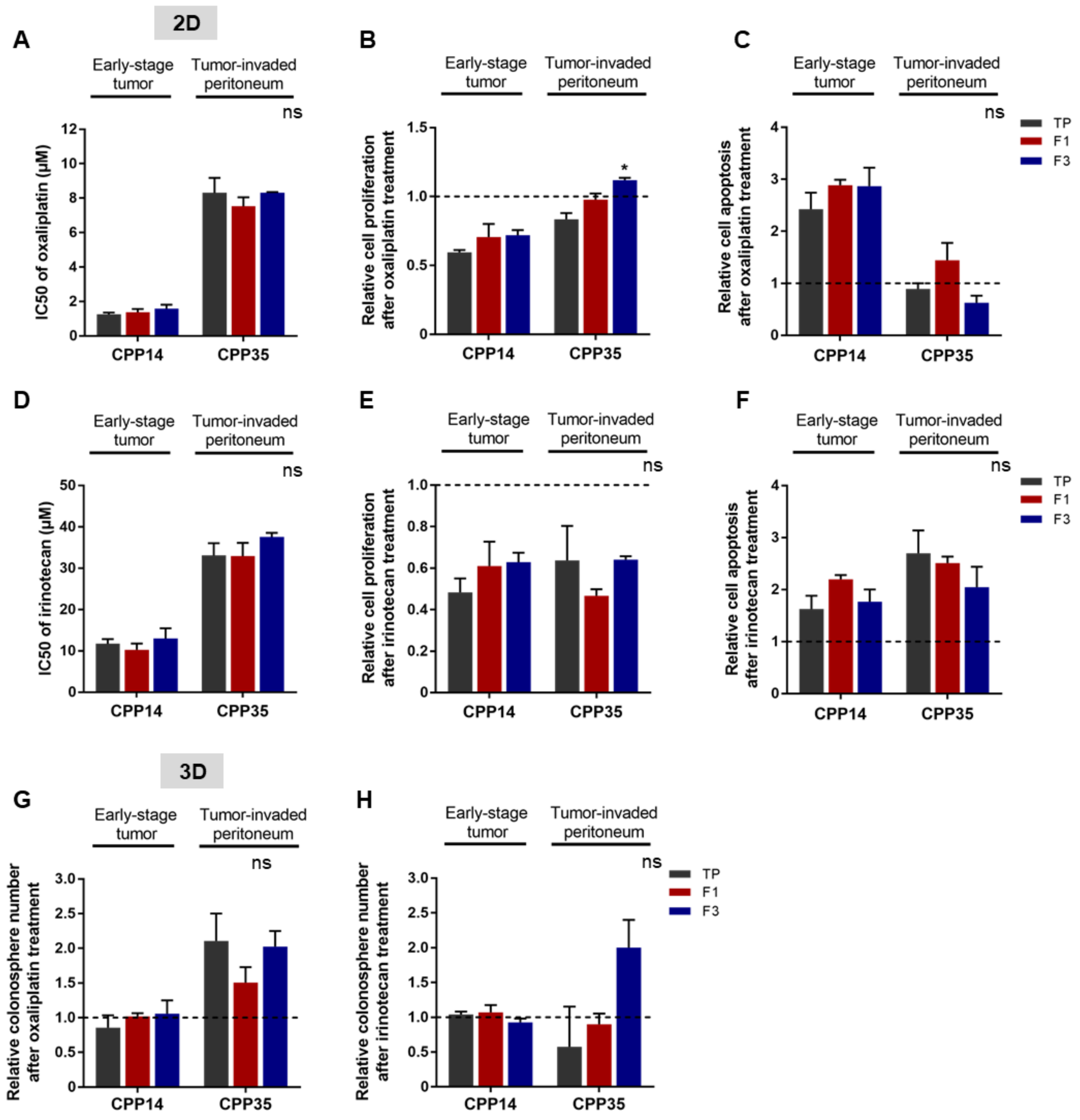

Figure S4. Response of SdFFF-sorted cell subpopulations to oxaliplatin and irinotecan from CRC primary cultures. (A-F) Response to oxaliplatin and irinotecan was assessed in 2D culture. (A-C) After oxaliplatin treatment, the evaluation of cell viability by MTT assay (A), cell proliferation by BrdU assay (B) and cell death by apoptosis by ELISA cell death (C) were performed. (D-F) Cell viability with IC50 values, proliferation and apoptosis were also evaluated after irinotecan treatment. (G-H) Response to oxaliplatin (G) and irinotecan (H) was also explored in 3D culture from colonospheres. All these results are represented as means ± SEM and statistical differences with ns means a non-significant result both between the sorted cell subpopulations and between a sorted subpopulation and the control, *p-value < 0.05 and *alone for significant results compared to TP using One-way ANOVA test.

Table S1. Lists of antibodies used for the analysis of CSC marker expression by flow cytometry.

Antibodies	Fluorescent dyes	References	Manufacturers
Anti-CD44 (G44-26)	FITC	555478	BD Pharmingen™
Anti-LGR5 (DA03-22H2.8)	PE-Vio 770	130-100-847	Miltenyi Biotec
Anti-CD133/1 (AC133)	PE-Vio 615	130-113-671	Miltenyi Biotec
Anti-BMI-1 (F-9)	PE	sc-390443	Santa Cruz

Viobility™ 405/452		130-109-816	Miltenyi Biotec
Anti-IgG2bκ (27-35)	FITC	555742	BD Pharmingen™
Anti-IgG2bκ (ES26-5E12.4)	PE-Vio 770	130-102-656	Miltenyi Biotec
Anti-IgG1 (IS5-21F5)	PE-Vio 615	130-113-201	Miltenyi Biotec
Anti-IgG1κ (MOPC-21)	PE	400111	Biolegend
